# Dynamics of torque teno virus load in kidney transplant recipients with indication biopsy and therapeutic modifications of immunosuppression

**DOI:** 10.3389/fmed.2024.1337367

**Published:** 2024-01-24

**Authors:** Marvin Reineke, Christian Morath, Claudius Speer, Markus Rudek, Christian Bundschuh, Julian A.F. Klein, Christoph F. Mahler, Florian Kälble, Christian Nusshag, Jörg Beimler, Martin Zeier, Ralf Bartenschlager, Paul Schnitzler, Louise Benning

**Affiliations:** ^1^Department of Nephrology, Heidelberg University Hospital, Heidelberg, Germany; ^2^German Center for Infection Research, DZIF, Heidelberg Partner Site, Heidelberg, Germany; ^3^Department of Molecular Medicine Partnership Unit Heidelberg, European Molecular Biology Laboratory, Heidelberg, Germany; ^4^Medical Faculty Heidelberg, Department of Infectious Diseases, Virology, Heidelberg University, Heidelberg, Germany; ^5^Medical Faculty Heidelberg, Department of Infectious Diseases, Molecular Virology, Center for Integrative Infectious Diseases Research, Heidelberg University, Heidelberg, Germany; ^6^Division Virus-Associated Carcinogenesis, German Cancer Research Center, Heidelberg, Germany

**Keywords:** kidney transplantation, immune monitoring, immunosuppression, torque teno virus, precision medicine

## Abstract

Following kidney transplantation, lifelong immunosuppressive therapy is essential to prevent graft rejection. On the downside, immunosuppression increases the risk of severe infections, a major cause of death among kidney transplant recipients (KTRs). To improve post-transplant outcomes, adequate immunosuppressive therapy is therefore a challenging but vital aspect of clinical practice. Torque teno virus load (TTVL) was shown to reflect immune competence in KTRs, with low TTVL linked to an elevated risk for rejections and high TTVL associated with infections in the first year post-transplantation. Yet, little is known about the dynamics of TTVL after the first year following transplantation and how TTVL changes with respect to short-term modifications in immunosuppressive therapy. Therefore, we quantified TTVL in 106 KTRs with 108 clinically indicated biopsies, including 65 biopsies performed >12 months post-transplantation, and correlated TTVL to histopathology. In addition, TTVL was quantified at 7, 30, and 90 days post-biopsy to evaluate how TTVL was affected by changes in immunosuppression resulting from interventions based on histopathological reporting. TTVL was highest in patients biopsied between 1 and 12 months post-transplantation (N = 23, median 2.98 × 10^7^ c/mL) compared with those biopsied within 30 days (N = 20, median 7.35 × 10^3^ c/mL) and > 1 year post-transplantation (N = 65, median 1.41 × 10^4^ c/mL; *p* < 0.001 for both). Patients with BK virus-associated nephropathy (BKVAN) had significantly higher TTVL than patients with rejection (*p* < 0.01) or other pathologies (*p* < 0.001). When converted from mycophenolic acid to a mTOR inhibitor following the diagnosis of BKVAN, TTVL decreased significantly between biopsy and 30 and 90 days post-biopsy (*p* < 0.01 for both). In KTR with high-dose corticosteroid pulse therapy for rejection, TTVL increased significantly between biopsy and 30 and 90 days post-biopsy (*p* < 0.05 and *p* < 0.01, respectively). Of note, no significant changes were seen in TTVL within 7 days of changes in immunosuppressive therapy. Additionally, TTVL varied considerably with time since transplantation and among individuals, with a significant influence of age and BMI on TTVL (*p* < 0.05 for all). In conclusion, our findings indicate that TTVL reflects changes in immunosuppressive therapy, even in the later stages of post-transplantation. To guide immunosuppressive therapy based on TTVL, one should consider inter- and intraindividual variations, as well as potential confounding factors.

## 1 Introduction

Lifelong immunosuppressive maintenance therapy is mandatory following kidney transplantation to prevent graft rejection and minimize risks for allograft failure (1, 2). However, immunosuppression increases the risk for severe infectious complications, which represent the leading non-cardiovascular cause of death among kidney transplant recipients (KTRs) (3–5). Therefore, optimal dosing of immunosuppressive drugs and balancing the risks of rejection and infection is important to improve outcomes after kidney transplantation.

Currently, monitoring of immunosuppression is mainly based on measuring calcineurin inhibitor trough levels, but acute rejection can occur even if trough levels are within the target range (6). Previously, Vasudev et al. introduced a semi-quantitative immunosuppression (IS) scale to assess the immunosuppressive burden in KTRs (7), which has been adopted and validated by various groups in different immunocompromised cohorts (8–11). However, as the IS scale is calculated using simply the dosages of immunosuppressive medication, it fails to address the high inter- and intrapatient variability in the dosage required to reach certain trough levels of immunosuppressive agents such as tacrolimus (12, 13). Thus, new surrogate parameters that measure a patient’s individual immunosuppressive burden are urgently needed to monitor immunocompetence.

Recently, monitoring torque teno virus (TTV) load within the first year post-transplantation has emerged as a promising approach to identify KTRs at risk of rejection or infection (14–17). Previous studies showed that TTV is profoundly influenced by the initiation of immunosuppressive therapy but largely evades the impact of antiviral drug therapy, such as cytomegalovirus prophylaxis administered after transplantation (18, 19). Additionally, a correlation has been observed between the intensity of immunosuppression and TTV load, indicating a potential link between TTV load and the likelihood of immunosuppression-related complications, including infections and rejections (14, 20). Recent data suggest that TTV loads may even help in monitoring short-term changes in immunosuppressive therapy, although there is still no thorough understanding of viral load kinetics due to changes in immunosuppression (21–23). To assess the possibility of guiding immunosuppression based on TTV loads in KTRs in the first year after transplantation, the multicentric, randomized, and controlled phase II TTVguideIT trial was initiated, with results expected in 2024 (24).

With limited data on the dynamics of TTV loads in KTRs transplanted >12 months ago, this study seeks to explore potential associations between TTV loads and various graft-associated pathologies, especially in KTRs beyond the first year post-transplantation. Additionally, this study aims to investigate changes in TTV loads upon modifications in immunosuppression in a well-characterized cohort of KTRs with indication biopsy at different time points post-transplantation.

## 2 Materials and methods

### 2.1 Study design

**Figure 1:**
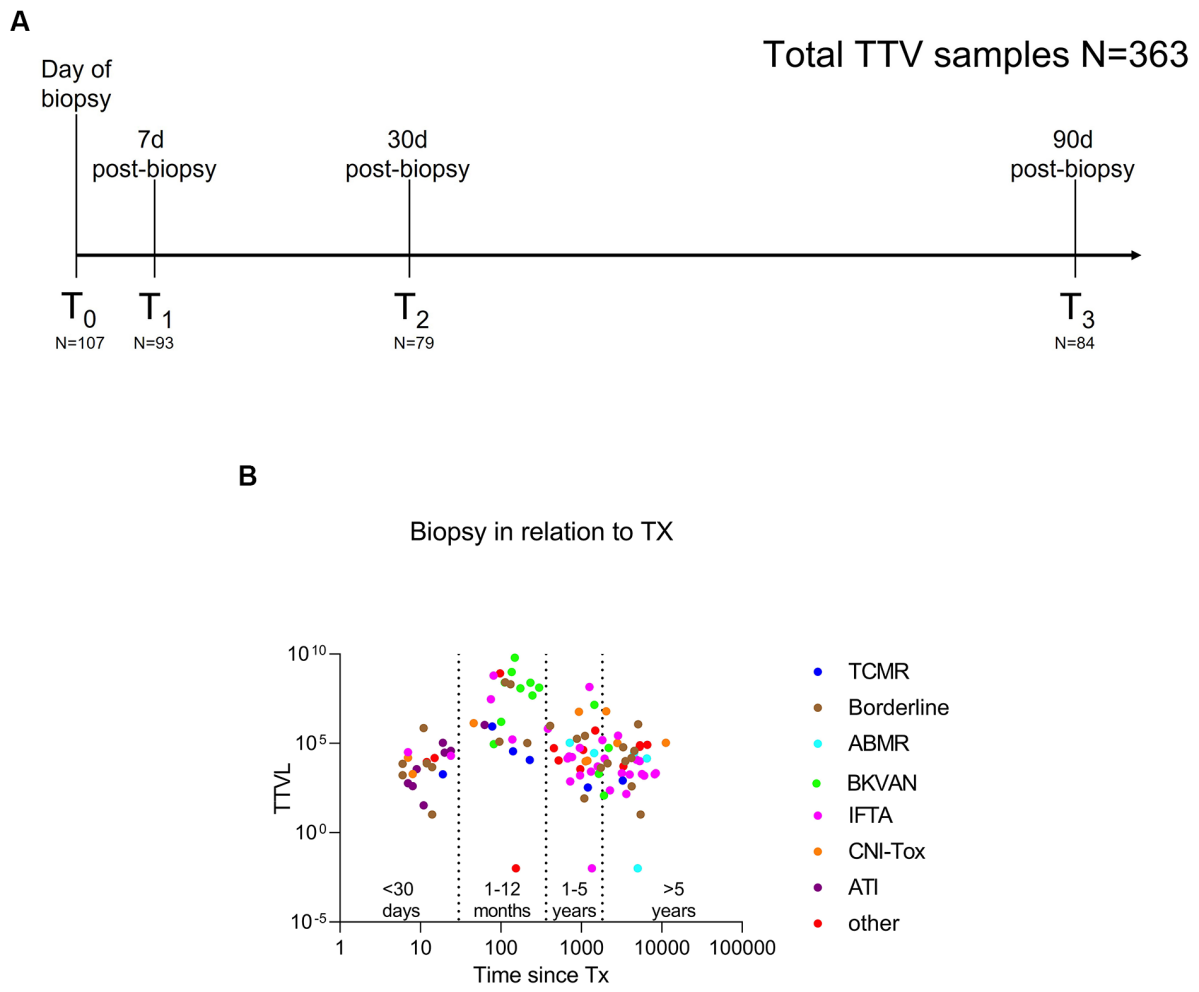
Study design to assess the dynamics of torque teno virus load in kidney transplant recipients with indication biopsy and therapeutic modifications of immunosuppression. **(A)** Torque teno virus load was quantified on the day of biopsy (T_0_, *N* = 107) and 7 days (T_1_, *N* = 93), 30 days (T_2_, *N* = 79) and 90 days (T_3_, *N* = 84) post-biopsy. In total, 363 patient samples were analyzed. **(B)** Torque teno virus load at biopsy (T_0_) in relation to timing of the biopsy relative to transplantation. Specifically, 20 KTRs received a biopsy within 30 days of transplantation, 23 KTRs received a biopsy between 1 and 12 months post-transplantation, and 65 KTRs underwent a biopsy >1 year post-transplantation. Different colors represent the different histopathological findings. 36/108 (33%) of indication biopsies were classified as rejection, including seven patients with ABMR (turquoise), six with TCMR (blue), and 23 with borderline changes (brown). BKVAN (green) was diagnosed in 13 KTRs. A total of 29 biopsies were graded as IFTA (pink), while 9 KTR showed signs of CNI toxicity (orange) and 8 presented ATI (purple). Other (red) diagnoses included eight patients with normal/unspecific histopathology, four KTR with recurrent disease, and one KTR with infect-related graft deterioration. ABMR, antibody-mediated rejection; ATI, acute tubular injury; BKVAN, BK virus-associated nephropathy; CNI, calcineurin inhibitor; IFTA, interstitial fibrosis and tubular atrophy; KTRs, kidney transplant recipients; TCMR, T cell-mediated rejection; TTVL, torque teno virus load; Tx, transplantation.

A total of 108 KTRs with indication biopsy at the Department of Nephrology, Heidelberg University Hospital, were enrolled in this prospective single-center study to evaluate new biomarkers in post-transplant care (DRKS00023604). Serum was obtained on the day of biopsy (T_0_), as well as 7 (T_1_), 30 (T_2_), and 90 days (T_3_) post-biopsy (Figure 1A), and TTV loads were quantified as a *post-hoc* analysis.

As immunosuppressive medication is reduced following initial transplantation and immunosuppressive burden consecutively declines with time since transplantation, KTRs were classified into four groups based on the timing of the biopsy in relation to initial transplantation (<30 days, *N* = 20; 1–12 months, *N* = 23; 1–5 years, *N* = 30; and > 5 years post-transplantation, *N* = 35) to compare TTV loads at the time of biopsy (T_0_) between these four groups. Biopsies were only performed in KTRs with no clinically apparent concomitant infection. Histopathology was assessed by two board-examined pathologists according to the BANFF 2018 reference guide (25), as reported previously (26).

Clinical management following histopathological reporting included corticosteroid pulse therapy in 31 patients with suspected rejection. Among the 13 KTRs with biopsy-proven BK virus-associated nephropathy (BKVAN), immunosuppression was switched from a calcineurin inhibitor (CNI)-mycophenolic acid (MPA) to a CNI-mTOR-based regimen including lower CNI target ranges. In six of these patients, MPA had already been reduced by 50% before biopsy due to the prior detection of BK viremia. Additionally, in six patients with suspected CNI toxicity (ah ≥ 1), immunosuppression with CNI was switched to belatacept. One of these patients further received corticosteroid pulse therapy for concomitant borderline lesions.

The study was approved by the ethics committee of the University of Heidelberg and conducted in accordance with the Declaration of Helsinki. All transplant procedures were performed in accordance with the Declaration of Istanbul. Written informed consent was obtained from all study participants. The main goals of this analysis were to: (i) assess differences in TTV loads between different graft-associated pathologies, particularly in KTRs transplanted >12 months ago; and (ii) analyze the effects of modifications in immunosuppressive therapy on TTV loads.

### 2.2 Quantification of torque teno virus load

TTV quantification was performed using the TTV R-Gene® assay (BioMérieux, Marcy-l’Etoile, France), a real-time polymerase chain reaction (PCR) assay targeting the TTV 5′ untranslated region (27). The assay has a dynamic range of 250 to 10^9^ copies/mL, with a limit of detection at 250 copies/mL. The assay was developed for quantifying TTV in plasma and whole blood samples, for which it is validated and widely used (28). We recently demonstrated that TTV load as quantified by the TTV R-Gene® assay did not differ significantly whether quantified in serum or plasma and observed a very strong and highly statistically significant correlation between serum and plasma TTV load, underscoring the interchangeability of serum and plasma for TTV quantification (21).

TTV DNA was extracted from serum samples using the QIAsymphony SP platform (QIAGEN, Venlo, the Netherlands), and PCR was conducted on a Light Cycler® 480 Instrument II (Roche Diagnostics, Rotkreuz, Switzerland) according to the manufacturer’s instructions. Viral load was determined using a standard curve, and specimens with undetectable viral load were assigned a value of 0.01 copies/mL for analysis purposes, as previously done by Fernández-Ruiz et al. (20).

### 2.3 Immunosuppression scale

To assess the overall burden of immunosuppression and to correlate results to TTV loads, an empiric, semi-quantitative score as previously published by Vasudev et al. was used (7). One score unit was assigned to each of the following doses of immunosuppressive drugs: tacrolimus 2 mg, cyclosporine 100 mg, mycophenolate mofetil 500 mg, azathioprine 100 mg, sirolimus 2 mg, and prednisolone 5 mg. For methylprednisolone and mycophenolate sodium, we assigned one unit to the equivalent doses of 4 mg and 360 mg, respectively. Additionally, we assigned one unit for 1.5 mg of everolimus, as previously done by Baumann et al. (29).

### 2.4 Statistical analysis

Quantitative data are presented as median with interquartile range (IQR). Due to TTV loads not conforming to normality, only non-parametric statistical analyses were performed. The Mann–Whitney *U* test or the Kruskal–Wallis test was used to compare continuous variables. To analyze repeated measures between pairs, the Wilcoxon matched-pairs rank test was applied. To correlate TTV load and IS score, medication, or BKV load, Spearman’s rho was calculated. The area under the ROC curve (AUC) was calculated to evaluate the performance of the TTV load to discriminate rejection from no rejection or BKVAN from no BKVAN. To identify possible confounders to TTV load, multiple linear regression was performed. For this analysis, TTV loads were log_10_-transformed beforehand. Statistical analysis was performed using GraphPad Prism version 9.5.1 (GraphPad Software, San Diego, CA, United States), and statistical significance was assumed at a *p*-value <0.05.

## 3 Results

### 3.1 Study cohort

Indication biopsy was performed at a median (IQR) of 2.6 (0.3–7.8) years post-transplantation, with 43/108 (40%) KTRs receiving a biopsy within the first year of transplantation. Specifically, 20 KTRs received a biopsy within 30 days of transplantation, 23 KTRs received a biopsy between 1 and 12 months post-transplantation, and 65 KTRs underwent a biopsy >1 year post-transplantation.

Histopathology revealed biopsy-proven rejection in 36 KTRs, including 7 KTRs with antibody-mediated rejection (ABMR), 6 KTRs with T-cell–mediated rejection (TCMR), and 23 KTRs with borderline changes. Patients with borderline changes were analyzed within the rejection group as all biopsies were performed on clinical indication. BKVAN (SV40+) was histopathologically proven in 13 KTRs. The other 59 KTRs were grouped as “No Rejection/BKVAN”, including 29 KTRs with interstitial fibrosis and tubular atrophy (IFTA), eight with acute tubular injury (ATI), nine with CNI toxicity, and 13 with other changes.

Figure 1B illustrates TTV loads in KTRs at the time of biopsy, considering the biopsy’s timing relative to time since transplantation, and color-coding representing the different histopathological diagnoses. Characteristics of the study cohort are presented in Table 1. Mean (±SD) age at biopsy was 49 (±14) years, 35 (32%) of the participants were female. Comorbidities and underlying renal pathologies for 108 KTRs with indication biopsy are shown in Supplementary Table S1.

**Table 1:** Characteristics of the study cohort.

Variable	All	Rejection	BKVAN	Other
Number of samples, *N*	108	36	13	59
Female, *N* (%)	35 (32)	12 (33)	4 (31)	19 (32)
Age at enrollment, mean ± SD	49 ± 14	46 ± 15	51 ± 11	51 ± 15
Donor type Deceased Donor, *N* (%) Living Donor, *N* (%)	70 (65) 38 (35)	16 (44) 20 (56)	10 (77) 3 (23)	44 (75) 15 (25)
HLA class 1 mismatches, mean ± SD	1.7 ± 1.2	1.9 ± 1.2	1.6 ± 1.2	1.6 ± 1.1
HLA class 2 mismatches, mean ± SD	0.8 ± 0.7	0.9 ± 0.7	0.8 ± 0.7	0.7 ± 0.6
Months post-transplant at time of biopsy, mean ± SD	60 ± 76	65 ± 73	22 ± 27	65 ± 84
DSA MFI > 500, *N* (%)	30 (29) ^+^	14 (39) ^++^	2 (15)	14 (24) ^+++^
DSA MFI > 1,000, *N* (%)	21 (20) ^+^	9 (25) ^++^	2 (15)	10 (17) ^+++^
S-Creatinine [mg/dl], mean ± SD	3.0 ± 2.3	2.9 ± 1.7	2.6 ± 1.2	3.1 ± 2.8
eGFR [ml/min/1.73 m^2^], mean ± SD	30.5 ± 16.3	30.0 ± 14.5	33.0 ± 19.5	30.4 ± 17.0
Proteinuria [g/molCr], mean ± SD	140.7 ± 217.2	209.6 ± 291.9	74.9 ± 115.4	114.6 ± 168.2
TTVL [copies/mL], median (IQR)	1.9 × 10^4^ (2.2 × 10^3^–2.7 × 10^5^)	1.4 × 10^4^ (1.9 × 10^3^–1.2 × 10^5^)	4.9 × 10^7^ (7.3 × 10^4^–2.5 × 10^8^)	1.5 × 10^4^ (2.2 × 10^3^–1.1 × 10^5^)
BKV load [IU/mL], median (IQR)			2.4 × 10^5^ (5.1 × 10^4^–6.9 × 10^5^)	

BKV, BK virus; BKVAN, BK virus-associated nephropathy; DSA, donor-specific antibodies; eGFR, estimated glomerular filtration rate; SD, standard deviation; TTVL, torque teno virus load.

^+^Not possible to determine DSA in five patients with missing data. ^++^not possible to determine DSA in two patients with missing data.

^+++^Not possible to determine DSA in three patients with missing data.

### 3.2 Torque teno virus prevalence and virus load kinetics according to timing of the biopsy

TTV was detectable in 107 (99%) of the study patients. Virus DNA was detectable in every sample in 104 patients, while three patients had one sample with a viral load below the threshold of detection. In total, 361 serum samples and 2 plasma samples were analyzed, with a mean of 3 samples analyzed per patient. TTV was quantifiable in 98.9% (359 of 363) of all samples with a median (IQR) viral load of 5.28 × 10^4^ c/mL (5.59 × 10^3^ c/mL–1.03× 10^6^ c/mL). TTV load varied markedly between patients, ranging from 10.3 c/mL to 7.44 × 10^9^ c/mL.

**Figure 2:**
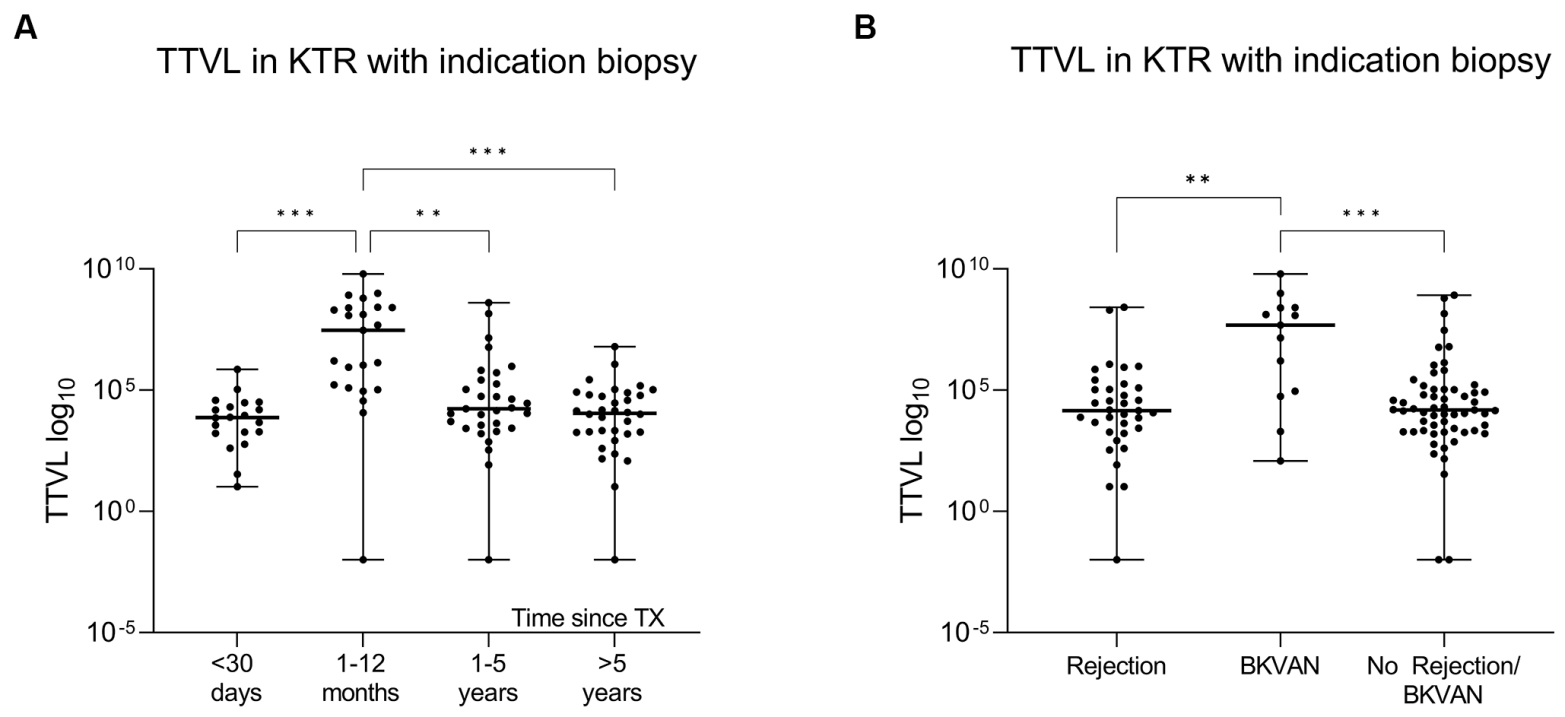
Differences in torque teno virus load based on time since transplantation and histopathology. **(A)** To compare TTVL at the time of biopsy (T_0_) in relation to time since transplantation, KTRs were categorized into four groups based on the timing of the biopsy relative to initial transplantation (<30 days, 1–12 months, 1–5 years and > 5 years post-transplantation). The x-axis displays the respective group, and the virus loads are shown on the y-axis. The scatter dot plots present the distribution of data with a horizontal line representing the median. The lower and upper edges display the minimum and maximum range, respectively. Individual values are shown as dots. **(B)** TTVL at time of biopsy (T_0_) in KTRs with rejection, BKVAN or other pathology. KTRs with BKVAN have significantly higher loads. The x-axis displays the respective group, and the virus loads are shown on the y-axis. The scatter dot plots present the distribution of data with a horizontal representing the median. The lower and upper edges display the minimum and maximum range, respectively. Individual values are shown as dots. BKVAN, BK virus-associated nephropathy; KTRs, kidney transplant recipients; TTVL, torque teno virus load; TX, transplantation; ***p < 0.001; **p < 0.01.

In patients who underwent indication biopsy less than 30 days post-transplantation, the median (IQR) TTV load was 7.35 × 10^3^ c/mL (1.71× 10^3^ c/mL–2.78 × 10^4^ c/mL). TTV load was with a median (IQR) of 2.98 × 10^7^ c/mL (1.24 × 10^5^ c/mL–2.58 × 10^8^ c/mL) significantly higher in patients that received a biopsy in between 1 and 12 months post-transplantation (*p* < 0.001). Subsequently, with a reduction in immunosuppression, TTV load was significantly lower in KTR with indication biopsies within 1 and 5 years (median 1.72 × 10^4^ c/mL, IQR 2.68 × 10^3^ c/mL–2.66 × 10^5^ c/mL, *p* = 0.001) and > 5 years post-transplantation (median 1.12 × 10^4^ c/mL, IQR 1.75 × 10^3^ c/mL–6.77 × 10^4^ c/mL; *p* < 0.001) compared with those with biopsies between 1 and 12 months post-transplantation (Figure 2A).

A multiple linear regression analysis revealed a significant influence of age, BMI, and time since transplantation on TTV loads (*p* < 0.05 for all; Supplementary Table S2).

### 3.3 Torque teno virus loads in kidney transplant recipients with different graft-associated pathologies

Patients with BKVAN had, with a median (IQR) of 4.85 × 10^7^ c/mL (7.34 × 10^5^ c/mL–2.54 × 10^8^ c/mL), significantly higher TTV loads at the time of biopsy than patients with histopathological signs of rejection (median 1.44 × 10^4^ c/mL, IQR 1.87 × 10^3^ c/mL–1.24 × 10^5^ c/mL) or other pathologies (median 1.51 × 10^4^ c/mL, IQR 2.17 × 10^3^ c/mL–1.06 × 10^5^ c/mL) (*p* < 0.01 and *p* < 0.001, respectively; Figure 2B). A total of two KTRs with rejection had a TTV load higher than the median TTV load for KTRs with BKVAN; these patients either had concurrent histoplasmosis or BK viremia at the time of biopsy. Additionally, three patients with no rejection or BKVAN showed higher TTV loads than the median TTV load for BKVAN. Of these three, two patients underwent indication biopsy within 3 to 4 months post-transplantation, a period where TTV loads in KTRs typically reach their peak levels, while the third patient was diagnosed with Pneumocystis jirovecii pneumonia shortly after biopsy.

**Figure 3:**
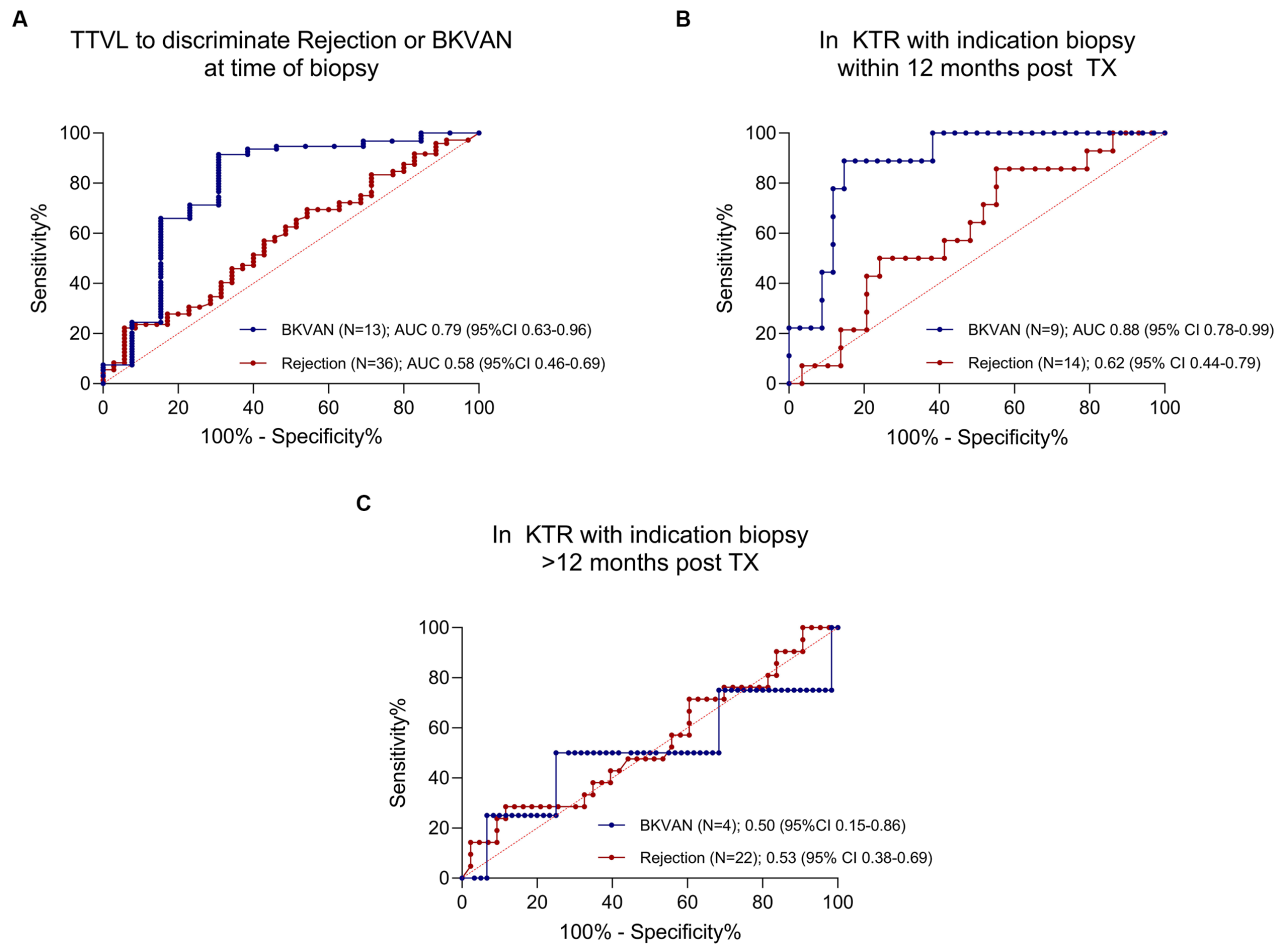
ROC curves for torque teno virus load to discriminate rejection and polyomavirus nephropathy from other diagnoses. **(A)** ROC curve for TTVL to discriminate rejection from no rejection and BKVAN from no BKVAN in all patients (*N* = 108). AUC for BKVAN = 0.79 (95% CI 0.63–0.96); AUC for rejection = 0.58 (95% CI 0.46–0.69). **(B)** ROC curve for TTVL to discriminate rejection from no rejection and BKVAN from no BKVAN in patients receiving a biopsy within the first year of transplantation (*N* = 43). AUC for BKVAN = 0.88 (95% CI 0.78–0.99); AUC for rejection = 0.62 (95% CI 0.44–0.79). **(C)** ROC curve for TTVL to discriminate rejection from no rejection and BKVAN from no BKVAN in KTRs receiving a biopsy after the first year of transplantation (*N* = 65). AUC for BKVAN = 0.50 (95% CI 0.15–0.86); AUC for rejection = 0.53 (95% CI 0.38–0.69). 100%-specificity % is displayed on the *x*-axis and sensitivity on the y-axis. The ROC curve to discriminate rejection is plotted in red whereas the ROC curve for BKVAN is plotted in blue. AUC, area under the curve; BKVAN, BK virus-associated nephropathy; CI, confidence interval; ROC, receiver operating characteristics; TTVL, torque teno virus load; TX, transplantation.

Considering all 108 KTRs with indication biopsy, the AUC to differentiate BKVAN from no BKVAN was at 0.79 (95% CI 0.63–0.96), and the AUC to discriminate rejection from no rejection was at 0.58 (95%CI 0.46–0.69; Figure 3A). When only including patients that received a biopsy within 1 year after transplantation (N = 43), the AUC to discriminate BKVAN from no BKVAN and rejection from no rejection increased to 0.88 (95% CI 0.78–0.99) and 0.62 (95% CI 0.44–0.79), respectively (Figure 3B). Figure 3C displays the ROC curves for KTRs that received a biopsy more than 1 year post-transplantation with an AUC of 0.50 (95% CI 0.15–0.86) to discriminate BKVAN from no BKVAN and an AUC of 0.53 (95% CI 0.38–0.69) to discriminate rejection from no rejection.

### 3.4 Influence of changes in immunosuppressive therapy on torque teno virus load

**Figure 4:**
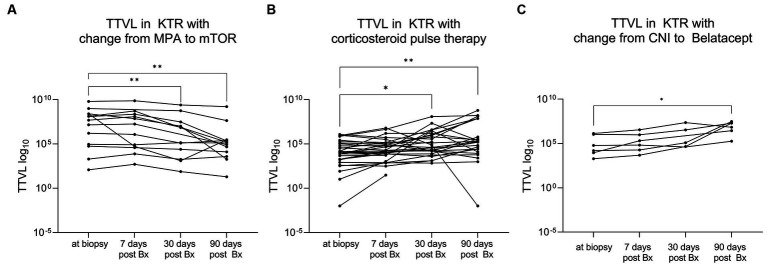
Dynamic changes in torque teno virus load following changes in immunosuppression. **(A)** KTRs with conversion from MPA to mTOR inhibitor following diagnosis for BKVAN (N = 13) showed a significant decrease in viral load. The *x*-axis displays the different time points of TTV sampling while TTVL is presented on the *y*-axis. The lines connect different samples of the same patient. **(B)** KTRs who received high-dose corticosteroid pulse therapy to treat rejection (N = 31) showed a significant increase in viral load. The *x*-axis displays the different time points while TTVL is presented on the y-axis. The lines connect different samples of the same patient. **(C)** KTRs who converted from CNI to belatacept (*N* = 6) showed a significant increase in TTVL. The x-axis displays the different time points while TTVL is presented on the y-axis. The lines connect different samples of the same patient. BKVAN, BK virus-associated nephropathy; Bx, biopsy; CNI, calcineurin inhibitor; KTRs, kidney transplant recipients; MPA, mycophenolic acid; mTOR, mammalian target of rapamycin; TTV, torque teno virus; TTVL, torque teno virus load; **p < 0.01; *p < 0.05.

When converted from mycophenolic acid (MPA) to the mTOR inhibitor following diagnosis for BKVAN (*N* = 13), TTV loads decreased significantly in these patients from a median (IQR) of 4.85 × 10^7^ c/mL (7.34 × 10^5^ c/mL–2.54 × 10^8^ c/mL) to a median (IQR) of 3.52 × 10^6^ c/mL (7.48 × 10^3^ c/mL–2.53 × 10^7^ c/mL) 30 days post-biopsy (T_2_; *p* < 0.01) and a median (IQR) of 1.32 × 10^5^ c/mL (8.33 × 10^3^ c/mL–2.73 × 10^5^ c/mL) 90 days post-biopsy (T_3_; *p* < 0.01; Figure 4A).

On the other side, in KTR who received high-dose corticosteroid pulse therapy as anti-rejection therapy (*N* = 31), a significant increase in TTV loads was observed between biopsy (T_0_) (median 1.17 × 10^4^ c/mL, IQR 1.66 × 10^3^ c/mL–1.03 × 10^5^ c/mL) to 30 days (T_2_) (median 7.53 × 10^4^ c/mL, IQR 1.14 × 10^4^ c/mL–1.34 × 10^6^ c/mL) and 90 days (T_3_) (median 1.83 × 10^5^ c/mL, IQR 1.89 × 10^4^ c/mL–3.72 × 10^7^ c/mL) post-biopsy (*p* < 0.05 and *p* < 0.01, respectively; Figure 4B).

Patients whose immunosuppressive therapy was converted from calcineurin inhibitors (CNI) to belatacept (*N* = 6) showed an increase in TTV loads as well, albeit less significant, with TTV loads surging from a median (IQR) of 3.78 × 10^4^ c/mL (7.36 × 10^3^ c/mL–1.15 × 10^6^ c/mL) at time of biopsy (T_0_) to a median (IQR) of 1.32 × 10^7^ c/mL (2.14 × 10^6^ c/mL–2.25 × 10^7^ c/mL) 90 days post-biopsy (T_3_; *p* < 0.05; Figure 4C).

### 3.5 Correlation of torque teno virus load to immunosuppressive medication, histopathological lesion score, and BK viremia

There was no significant correlation between TTV load and time since transplantation or IS scale when considering all KTR, irrespective of the timing of the biopsy (*r* = −0.17 and *r* = 0.03, respectively; Table 2). When only including KTR with an indication biopsy at least 30 days post-transplantation whose viral loads had already increased to higher levels post-transplantation (N = 84), TTV load correlated significantly and strongly with time since transplantation and moderately with IS scale (*r* = −0.53, *p* < 0.001 and *r* = 0.28, *p* < 0.01, respectively; Table 2). When examining the correlation between single immunosuppressants to TTV load, only an immunosuppressive regimen including everolimus showed a significant, negative correlation to TTV load (*r* = −0.20, *p* < 0.05; Table 3).

**Table 2:** Correlation between torque teno virus load, immunosuppression scale, and time since transplantation.

Variable	All KTR (*N* = 103) ^+^	KTR with biopsy > 30 days post-Tx (*N* = 84) ^++^
IS Scale: median (IQR)	6.0 (4.0–9.5)	5.0 (4.0–7.0)

Correlation	Spearman’s rho (95% CI)	*p* value	Spearman’s rho (95% CI)	*P* value
TTVL/Time since Tx	-0.17 (-0.36–0.03)	0.09	-0.53 (-0.67– -0.35)	<0.001 (***)
IS Scale/TTVL	0.03 (-0.17–0.23)	0.76	0.28 (0.06–0.47)	0.01 (**)
IS Scale/Time since Tx	-0.74 (-0.82– -0.64)	<0.001 (***)	-0.55 (-0.68– -0.37)	<0.001 (***)

CI, confidence interval; IS, immunosuppression; KTR, kidney transplant recipients; TTVL, torque teno virus load; Tx, transplantation.

^+^five patients whose immunosuppressive regimen at biopsy included belatacept were excluded from the analysis ^++^ four patients whose immunosuppressive regimen at biopsy included belatacept were excluded from the analysis.

**Table 3:** Immunosuppressive medication and correlation to torque teno virus loads.

Immunosuppressive regimen: *N* (%)	All	Spearman’s rho (95% CI)	*P*-value
Tacrolimus	82 (76)	0.17 (-0.02–0.36)	0.07
Cyclosporine	19 (18)	-0.09 (-0.28–0.11)	0.35
Mycophenolic acid	91 (84)	0.03 (-0.17–0.22)	0.78
Azathioprine	3 (3)	-0.08 (-0.27–0.11)	0.40
Everolimus	8 (7)	-0.20 (-0.38–0.00)	0.04 (*)
Sirolimus	1 (1)	-0.09 (-0.28–0.11)	0.38
Belatacept	5 (5)	0.00 (-0.20–0.19)	0.97
Corticosteroid	103 (95)	0.13 (-0.07–0.32)	0.18

CI, confidence interval; *P < 0.05.

When analyzing the correlation between TTV loads at the time of biopsy and histopathological BANFF lesion scores, TTV load correlated weakly to BANFF lesion score for inflammation (i; *r* = 0.23, *p* < 0.05) and moderately to polyomavirus-associated interstitial nephritis score (PVI, *r* = 0.35, *p* < 0.001), while there was no significant correlation to other BANFF lesion scores (Supplementary Table S3).

In KTR with BKVAN, there was a moderate correlation between BKV loads and TTV loads, with higher BK viremia being associated with higher TTV load (*r* = 0.36, *p* < 0.05). Following conversion from MPA to mTOR inhibitor (N = 13), a notable reduction in BKV loads was observed when comparing levels at 30 and 90 days post-biopsy to BKV loads 7 days post-biopsy (*p* = 0.01 for both, Supplementary Figure S1). Of thirteen, three KTR (23%) demonstrated an increase in BK viremia, despite a concurrent decrease in TTV loads. When comparing BKV and TTV loads at time of biopsy to those documented 90 days post-biopsy, there was no significant correlation between the delta viral loads (*p* = 0.57).

## 4 Discussion

Our results validate the potential of quantifying TTV loads to monitor immunocompetence in KTR within the first year post-transplantation. However, it appears to be challenging to correctly identify patients with borderline lesions suspicious for TCMR even within the first year post-transplantation, as well as differentiating between KTR with BKVAN or rejection and those without beyond 12 months post-transplantation, solely based on TTV loads. On another note, we show that TTV loads mirror adjustments in immunosuppressive therapy, albeit TTV loads do not seem to be affected immediately (within 7 days) following corticosteroid pulse therapy or switching immunosuppression to an mTOR-based regimen.

Our data demonstrate that KTR with BKVAN had significantly higher TTV loads compared with KTR with rejection or other pathologies, which was also reflected by a moderate correlation between TTV loads to histopathological SV40 positivity. In addition, higher BKV loads were associated with higher TTV loads in KTR with BK viremia, as previously shown by Solis et al. (30). Higher TTV loads may reflect a higher immunosuppressive burden, possibly fueling the replication of BK virus and the potential development of BKVAN. While a general association between TTV loads and BK viremia in the first 3 months post-transplantation may not be evident (31), possibly due to other confounding factors such as induction therapy, our findings suggest that higher TTV loads could help in identifying patients with risk for BKVAN, especially in the first year post-transplantation.

On the other hand, KTRs with active rejection had significantly lower TTV loads compared with KTRs with BKVAN; however, no significant differences were seen between KTRs with histopathologically diagnosed rejection and those with histopathology other than rejection or BKVAN. This could be attributed to the substantial number of patients with borderline lesions (*N* = 23) among those with rejection (*N* = 36). Despite the ongoing controversy regarding the pathological relevance and the need for treatment of borderline lesions, previous studies have associated these lesions with adverse outcomes such as late rejection, functional impairment, donor-specific antibody formation, and allograft failure (32), which would advocate for the necessity to timely detect KTRs with those lesions.

Jaksch et al. proposed an optimal TTV load range of 4.6 log_10_ c/mL to 6.6 log_10_ c/mL for KTR within 3 months and 1 year post-transplantation that strikes a balance between risks for rejection and infection (33). In our study cohort, there were 17 KTRs within this time frame: of the eight patients, seven (88%) with BKVAN were above the proposed upper cut-off indicating an elevated risk for infection (*p* < 0.05). Applying the lower threshold to identify patients at risk for rejection however only correctly diagnosed two patients with active TCMR, while all four patients with borderline changes had TTV loads above the threshold and would have been missed (*p* = 0.51). Admittedly, the small sample size (*N* = 17) to whom the cut-offs were applicable in our cohort limits a definitive conclusion; however, the proposed cut-offs by Jaksch et al. appear to be promising indicators for infection but seem to fail to early identify patients with borderline changes suspicious for TCMR. Corresponding to that, the AUC of 0.62 to discriminate rejection from no rejection in KTRs that were biopsied within 1 year post-transplantation in our study cohort was rather weak and not conclusive.

The results to correctly identify rejection or BKVAN beyond 12 months post-transplantation were rather disappointing. This may be attributed to the fact that we noted significant variations in TTV loads depending on the timing of the indication biopsy relative to transplantation, even beyond the first year post-transplantation, which is consistent with previous research (34, 35). Furthermore, we revealed a significant association between higher TTV loads and age, which has been demonstrated before for healthy adults (36, 37) as well as for KTRs (20, 34), and may be explained by immunosenescence and the accompanying higher susceptibility to pathogens in the elderly population (38). Moreover, a higher BMI was linked to elevated TTV loads, suggesting impaired immune functionality in obese patients compared with non-obese individuals (39).

Of note, TTV loads reflected changes in immunosuppressive therapy within our study. KTRs switching to mTOR inhibitors following diagnosis for BKVAN displayed a notable decrease in TTV loads, consistent with the negative correlation we found between mTOR inhibitor intake and TTV loads. Our findings align with results obtained by Schiemann et al., revealing significantly reduced TTV levels in KTRs under mTOR inhibitor-based maintenance therapy (34). This effect may reflect the antiviral properties of mTOR inhibitors but possibly also the generally reduced efficacy compared with CNIs (40). Contrarily, patients receiving high-dose corticosteroid pulse therapy for rejection subsequently developed significantly higher TTV loads. This aligns with de Vlaminck et al. who reported that higher prednisone doses early after transplantation result in an increased presence of TTV (18). Our results add that TTV loads could additionally serve as an indicator of the higher immunosuppressive burden in KTRs receiving anti-rejection therapy beyond the first year following transplantation. Furthermore, our data support the hypothesis that changes in TTV load reflect modifications in immunosuppression, as previously shown for short-time cessation of mycophenolate in KTRs (21, 22). Interestingly, the decline in TTV loads observed in our patients with BKVAN transitioning from MPA to an mTOR-based regimen did not consistently align with a reduction in BK viremia, preventing the utilization of TTV loads as a means to monitor treatment response in individuals with BK viremia. Additionally, further investigation is needed to determine the extent and pace of changes in TTV loads as a response to modifications in CNI dosages, as well as the potential utility of TTV in guiding CNI dosage adjustments (24).

It seems evident that simple tools such as the immunosuppression scale proposed by Vasudev et al. in 2005 to evaluate the degree of immunosuppression in KTRs with BKVAN (7) seem insufficient to monitor both, infections and rejections post-transplantation. Within our cohort, TTV loads correlated more strongly to time since transplantation than to the IS scale, emphasizing that a mere scoring system for various immunosuppressants does not sufficiently capture the true extent of immunosuppression in a patient. Our data suggest that TTV loads are more accurate than the IS scale or drug trough levels in reflecting the immunosuppressive burden in immunocompromised patients and may prove particularly useful for monitoring complications related to over- or under immunosuppression in older and obese patients.

In general, our study has some limitations. First, the single-center design compromises external generalization. Second, the small sample size of some sub-analyses may limit their validity. All proposed correlations should therefore merely be considered as hypothesis-generating, emphasizing the need for further studies. Additionally, as no re-biopsies were performed within this study cohort, it was not possible to establish a correlation between TTV loads and potential histopathological resolution of injury in patients experiencing rejections or BKVAN.

In conclusion, we were able to reproduce previous findings that TTV loads are highest in patients within the first year after transplantation and gradually become lower in patients transplanted long ago, corresponding to a reduction in immunosuppression following transplantation. To guide immunosuppressive therapy based on TTV loads, one should consider inter- and intraindividual variations, as well as confounding factors such as age, BMI, and, most importantly, time since transplantation, even beyond the first year post-transplantation. Another potential use case of monitoring TTV loads could be to follow up on changes in immunosuppressive therapy, although viral replication does not appear to be immediately impacted following corticosteroid pulse therapy or the transition to an mTOR-based immunosuppressive regimen.

## Data availability statement

The raw data supporting the conclusions of this article will be made available by the authors, without undue reservation.

## Ethics statement

The studies involving humans were approved by the Ethikkommission der Medizinischen Fakultät Heidelberg. The studies were conducted in accordance with the local legislation and institutional requirements. The participants provided their written informed consent to participate in this study.

## Author contributions

MRe: Data curation, Formal analysis, Investigation, Methodology, Visualization, Writing – original draft. CMo: Funding acquisition, Resources, Supervision, Validation, Writing – review & editing. CS: Validation, Writing – review & editing. MRu: Data curation, Writing – review & editing. CB: Investigation, Methodology, Supervision, Writing – review & editing. JK: Investigation, Methodology, Supervision, Writing – review & editing. CMa: Data curation, Writing – review & editing. FK: Data curation, Writing – review & editing. CN: Data curation, Writing – review & editing. JB: Data curation, Writing – review & editing. MZ: Data curation, Funding acquisition, Project administration, Resources, Supervision, Writing – review & editing. RB: Methodology, Project administration, Resources, Supervision, Writing – review & editing. PS: Data curation, Methodology, Project administration, Resources, Supervision, Writing – review & editing. LB: Conceptualization, Data curation, Formal analysis, Funding acquisition, Investigation, Methodology, Project administration, Resources, Supervision, Validation, Visualization, Writing – original draft.

## References

[1] Sellarés J de Freitas DG Mengel M Reeve J Einecke G Sis B. Understanding the causes of kidney transplant failure: the dominant role of antibody-mediated rejection and nonadherence. Am J Transplant. (2012) 12:388–99. doi: 10.1111/j.1600-6143.2011.03840.x, PMID: 22081892

[2] Betjes MGH Roelen DL van Agteren M Kal-van Gestel J. Causes of kidney graft failure in a cohort of recipients with a very long-time follow-up after transplantation. Front Med. (2022) 9:842419. doi: 10.3389/fmed.2022.842419, PMID: 35733857 PMC9207199

[3] van Delden C Stampf S Hirsch HH Manuel O Meylan P Cusini A. Burden and timeline of infectious diseases in the first year after solid organ transplantation in the Swiss transplant cohort study. Clin Infect Dis. (2020) 71:e159–69. doi: 10.1093/cid/ciz1113, PMID: 31915816 PMC7583409

[4] Farrugia D Cheshire J Begaj I Khosla S Ray D Sharif A. Death within the first year after kidney transplantation – an observational cohort study. Transpl Int. (2014) 27:262–70. doi: 10.1111/tri.12218 24138318

[5] Fishman JA. Infection in organ transplantation. Am J Transplant. (2017) 17:856–79. doi: 10.1111/ajt.14208 28117944

[6] Andrews LM Li Y Winter BCMD Shi Y-Y Baan CC Gelder TV. Pharmacokinetic considerations related to therapeutic drug monitoring of tacrolimus in kidney transplant patients. Expert Opin Drug Metab Toxicol. (2017) 13:1225–36. doi: 10.1080/17425255.2017.1395413, PMID: 29084469

[7] Vasudev B Hariharan S Hussain SA Zhu Y-R Bresnahan BA Cohen EP. BK virus nephritis: risk factors, timing, and outcome in renal transplant recipients. Kidney Int. (2005) 68:1834–9. doi: 10.1111/j.1523-1755.2005.00602.x, PMID: 16164661

[8] Assawasaksakul T Lertussavavivat T Sathitratanacheewin S Oudomying N Vichaiwattana P Wanlapakorn N. Comparison of immunogenicity and safety of inactivated, adenovirus-vectored, and heterologous adenovirus-vectored/mRNA vaccines in patients with systemic lupus erythematosus and rheumatoid arthritis: a prospective cohort study. Vaccine. (2022) 10:853. doi: 10.3390/vaccines10060853, PMID: 35746461 PMC9227480

[9] Shaked A Des Marais MR Kopetskie H Feng S Punch JD Levitsky J. Outcomes of immunosuppression minimization and withdrawal early after liver transplantation. Am J Transplant. (2019) 19:1397–409. doi: 10.1111/ajt.15205, PMID: 30506630 PMC6482056

[10] Engel B Görzer I Campos-Murguia A Hartleben B Puchhammer-Stöckl E Jaeckel E. Association of torque Teno virus viremia with liver fibrosis in the first year after liver transplantation. Front Immunol. (2023) 14:1215868. doi: 10.3389/fimmu.2023.1215868, PMID: 37533865 PMC10392936

[11] Saidy RRO Wegener E Uluk D Dittrich L Schöning W Lurje G. A reduction of Calcineurin inhibitors may improve survival in patients with De novo colorectal Cancer after liver transplantation. Medicina. (2022) 58:1755. doi: 10.3390/medicina58121755, PMID: 36556957 PMC9785597

[12] Van GT. Drug interactions with tacrolimus. Drug Saf. (2002) 25:707–12. doi: 10.2165/00002018-200225100-00003 12167066

[13] Shuker N Van GT Hesselink DA. Intra-patient variability in tacrolimus exposure: causes, consequences for clinical management. Transplant Rev. (2015) 29:78–84. doi: 10.1016/j.trre.2015.01.002 25687818

[14] Strassl R Schiemann M Doberer K Görzer I Puchhammer-Stöckl E Eskandary F. Quantification of torque Teno virus viremia as a prospective biomarker for infectious disease in kidney allograft recipients. J Infect Dis. (2018) 218:1191–9. doi: 10.1093/infdis/jiy306, PMID: 30007341 PMC6490304

[15] Strassl R Doberer K Rasoul-Rockenschaub S Herkner H Görzer I Kläger JP. Torque Teno virus for risk stratification of acute biopsy-proven alloreactivity in kidney transplant recipients. J Infect Dis. (2019) 219:1934–9. doi: 10.1093/infdis/jiz039, PMID: 30668796 PMC6534191

[16] Doberer K Schiemann M Strassl R Haupenthal F Dermuth F Görzer I. Torque Teno virus for risk stratification of graft rejection and infection in kidney transplant recipients—a prospective observational trial. Am J Transplant. (2020) 20:2081–90. doi: 10.1111/ajt.15810, PMID: 32034850 PMC7496119

[17] Doberer K Haupenthal F Nackenhorst M Bauernfeind F Dermuth F Eigenschink M. Torque Teno virus load is associated with subclinical alloreactivity in kidney transplant recipients: a prospective observational trial. Transplantation. (2021) 105:2112–8. doi: 10.1097/tp.0000000000003619, PMID: 33587432 PMC8376270

[18] De Vlaminck I Khush KK Strehl C Kohli B Luikart H Neff NF. Temporal response of the human Virome to immunosuppression and antiviral therapy. Cell. (2013) 155:1178–87. doi: 10.1016/j.cell.2013.10.034, PMID: 24267896 PMC4098717

[19] Moen EM Sagedal S Bjøro K Degré M Opstad PK Grinde B. Effect of immune modulation on TT virus (TTV) and TTV-like-mini-virus (TLMV) viremia. J Méd Virol. (2003) 70:177–82. doi: 10.1002/jmv.10356, PMID: 12629661

[20] Fernández-Ruiz M Albert E Giménez E Ruiz-Merlo T Parra P López-Medrano F. Monitoring of alphatorquevirus DNA levels for the prediction of immunosuppression-related complications after kidney transplantation. Am J Transplant. (2019) 19:1139–49. doi: 10.1111/ajt.15145, PMID: 30346659

[21] Benning L Reineke M Bundschuh C Klein JAF Kühn T Zeier M. Quantification of torque Teno virus load to monitor short-term changes in immunosuppressive therapy in kidney transplant recipients. Transplantation. (2023) 107:e363–9. doi: 10.1097/tp.0000000000004816, PMID: 37798825

[22] Regele F Heinzel A Hu K Raab L Eskandary F Faé I. Stopping of mycophenolic acid in kidney transplant recipients for 2 weeks Peri-vaccination does not increase response to SARS-CoV-2 vaccination—a non-randomized, controlled pilot study. Front Med. (2022) 9:914424. doi: 10.3389/fmed.2022.914424, PMID: 35755078 PMC9226446

[23] Haupenthal F Bond G. Torque Teno viral plasma load for immunologic monitoring in solid organ transplantation: one step further. Transplantation. (2023) 107:e326–7. doi: 10.1097/tp.0000000000004817, PMID: 37798831 PMC10664785

[24] Haupenthal F Rahn J Maggi F Gelas F Bourgeois P Hugo C. A multicentre, patient-and assessor-blinded, non-inferiority, randomised and controlled phase II trial to compare standard and torque Teno virus-guided immunosuppression in kidney transplant recipients in the first year after transplantation: TTVguideIT. Trials. (2023) 24:213. doi: 10.1186/s13063-023-07216-0, PMID: 36949445 PMC10032258

[25] Roufosse C Simmonds N Groningen MC Haas M Henriksen KJ Horsfield C. A 2018 reference guide to the Banff classification of renal allograft pathology. Transplantation. (2018) 102:1795–814. doi: 10.1097/tp.0000000000002366, PMID: 30028786 PMC7597974

[26] Benning L Morath C Fink A Rudek M Speer C Kälble F. Donor-derived cell-free DNA (dd-cfDNA) in kidney transplant recipients with indication biopsy—results of a prospective single-center trial. Transpl Int. (2023) 36:11899. doi: 10.3389/ti.2023.11899, PMID: 38020751 PMC10654198

[27] Focosi D Antonelli G Pistello M Maggi F. Torquetenovirus: the human virome from bench to bedside. Clin Microbiol Infec. (2016) 22:589–93. doi: 10.1016/j.cmi.2016.04.007, PMID: 27093875

[28] Kulifaj D Durgueil-Lariviere B Meynier F Munteanu E Pichon N Dubé M. Development of a standardized real time PCR for torque Teno viruses (TTV) viral load detection and quantification: a new tool for immune monitoring. J Clin Virol. (2018) 105:118–27. doi: 10.1016/j.jcv.2018.06.010, PMID: 29957546

[29] Baumann AK Schlue J Noyan F Hardtke-Wolenski M Lehner F Barg-Hock H. Preferential accumulation of T helper cells but not cytotoxic T cells characterizes benign subclinical rejection of human liver allografts. Liver Transplant. (2016) 22:943–55. doi: 10.1002/lt.24427 26929119

[30] Solis M Velay A Gantner P Bausson J Filipputtu A Freitag R. Torquetenovirus viremia for early prediction of graft rejection after kidney transplantation. J Infect. (2019) 79:56–60. doi: 10.1016/j.jinf.2019.05.010, PMID: 31100359

[31] Handala L Descamps V Morel V Castelain S François C Duverlie G. No correlation between torque Teno virus viral load and BK virus replication after kidney transplantation. J Clin Virol. (2019) 116:4–6. doi: 10.1016/j.jcv.2019.03.018, PMID: 30986626

[32] Nankivell BJ. The meaning of borderline rejection in kidney transplantation. Kidney Int. (2020) 98:278–80. doi: 10.1016/j.kint.2020.04.052, PMID: 32709286

[33] Jaksch P Görzer I Puchhammer-Stöckl E Bond G. Integrated immunologic monitoring in solid organ transplantation: the road toward torque Teno virus-guided immunosuppression. Transplantation. (2022) 106:1940–51. doi: 10.1097/tp.0000000000004153 35509090 PMC9521587

[34] Schiemann M Puchhammer-Stöckl E Eskandary F Kohlbeck P Rasoul-Rockenschaub S Heilos A. Torque Teno virus load—inverse association with antibody-mediated rejection after kidney transplantation. Transplantation. (2017) 101:360–7. doi: 10.1097/tp.0000000000001455 27525643 PMC5268087

[35] Gore EJ Gomes-Neto AW Wang L Bakker SJL Niesters HGM De JAAE. Torquetenovirus serum load and long-term outcomes in renal transplant recipients. J Clin Med. (2020) 9:440. doi: 10.3390/jcm9020440, PMID: 32041187 PMC7073853

[36] Brassard J Gagné M-J Leblanc D Poitras É Houde A Boras VF. Association of age and gender with torque Teno virus detection in stools from diarrheic and non-diarrheic people. J Clin Virol. (2015) 72:55–9. doi: 10.1016/j.jcv.2015.08.020, PMID: 26401905

[37] Haloschan M Bettesch R Görzer I Weseslindtner L Kundi M Puchhammer-Stöckl E. TTV DNA plasma load and its association with age, gender, and HCMV IgG serostatus in healthy adults. Age. (2014) 36:9716. doi: 10.1007/s11357-014-9716-2, PMID: 25284090 PMC4185385

[38] Lee K-A Flores RR Jang IH Saathoff A Robbins PD. Immune senescence, Immunosenescence and aging. Front Aging. (2022) 3:900028. doi: 10.3389/fragi.2022.900028, PMID: 35821850 PMC9261375

[39] Herz CT Kulterer OC Kulifaj D Gelas F Franzke B Haupenthal F. Obesity is associated with a higher torque Teno viral load compared to leanness. Front Endocrinol. (2022) 13:962090. doi: 10.3389/fendo.2022.962090, PMID: 36246898 PMC9554490

[40] Bowman LJ Brueckner AJ Doligalski CT. The role of mTOR inhibitors in the Management of Viral Infections. Transplantation. (2018) 102:S50–9. doi: 10.1097/tp.0000000000001777 29369973

