# Supplementary material for: Dynamics of torque teno virus load in kidney transplant recipients with indication biopsy and therapeutic modifications of immunosuppression
Source: Front Med (Lausanne). 2024 Jan 24;11:1337367. doi: 10.3389/fmed.2024.1337367 (PMC10847215; doi:10.3389/fmed.2024.1337367)
Supplement: Supplementary file 1 [file Data_Sheet_1.PDF]

Supplementary Material

Table of Contents

Supplementary Table S1 Comorbidities and underlying renal pathology in 108 kidney transplant recipients with indication biopsy.

Supplementary Table S2 Multiple linear regression analysis to identify possible confounders to torque teno virus load.

Supplementary Table S3 Correlation between BANFF lesion score, the polyomavirus-associated interstitial nephritis score and torque teno virus load.

Supplementary Figure S1 Dynamic changes in BK virus load following conversion from mycophenolic acid to mTOR inhibition in 13 kidney transplant recipients with BK virus-associated nephropathy.

Supplementary Tables

Supplementary Table S1 Comorbidities and underlying renal pathology in 108 kidney transplant recipients with indication biopsy.

Comorbidities: N (%)	All<br>N=108	Rejection<br>N=36	BKVAN<br>N=13	Other<br>N=59
Hypertension	90 (83)	28 (78)	12 (92)	50 (85)
Diabetes	16 (15)	2 (6)	3 (23)	11 (19)
Coronary artery disease	28 (26)	8 (22)	5 (38)	15 (25)
Chronic lung disease	14 (13)	7 (19)	1 (8)	6 (10)
Chronic liver disease	9 (8)	1 (3)	2 (15)	6 (10)
Malignancy	6 (6)	4 (11)	0 (0)	2 (3)
**Underlying renal pathology: N (%)**				
Glomerulonephritis	49 (45)	13 (36)	5 (38)	31 (53)
Diabetic nephropathy	6 (6)	1 (3)	1 (8)	4 (7)
Interstitial nephritis	6 (6)	4 (11)	0 (0)	2 (3)
Vascular	9 (8)	3 (8)	1 (8)	5 (8)
Polycystic Kidney Disease	13 (12)	4 (11)	2 (15)	7 (12)
Systemic disease	4 (4)	2 (6)	1 (8)	1 (2)
Other	17 (16)	7 (19)	2 (15)	8 (14)
Unknown	4 (4)	2 (6)	1 (8)	1 (2)

BKVAN, BK virus-associated nephropathy

Supplementary Table S2 Multiple linear regression analysis to identify possible confounders to torque teno virus load^{+}.

Characteristic	B	SE	95% CI	P value
Age	0.027	0.014	8.9×10^{-5}–0.055	0.05 (*)
Gender	-0.203	0.453	-1.103–0.698	0.66
BMI	0.073	0.028	0.018–0.129	0.01 (*)
Time since Tx	-0.007	0.003	-0.012–-0.001	0.03 (*)
eGFR	0.023	0.013	-0.002–0.048	0.08
Rejection including Borderline	0.718	0.480	-0.235–1.671	0.14
DSA MFI>500	0.655	0.798	-0.930–2.241	0.41
DSA MFI>1000	0.085	0.887	-1.675–1.846	0.92

B, regression coefficient; BMI, body mass index; CI, confidence interval of regression coefficient B; DSA, donor-specific antibodies; MFI, mean fluorescence intensity; SE, standard error; TX, transplantation. $^{+}$ TTV loads were log10-transformed for analysis.

Supplementary Table S3 Correlation between BANFF lesion score, the polyomavirus-associated interstitial nephritis score and torque teno virus load.

Lesion Score	Biopsies with detectable lesion, N (%)	Spearman's rho (95% CI)	P value
Interstitial inflammation (i)	66 (61)	0.23 (0.03–0.41)	0.02 (*)
Tubulitis (t)	44 (41)	0.17 (-0.03–0.36)	0.08
Intimal arteritis (v)	1 (1)	0.11 (-0.10–0.31)	0.28
Glomerulitis (g)	1 (1)	-0.16 (-0.35–0.04)	0.11
Peritubular capillaritis (ptc)	44 (41)	-0.12 (-0.32–0.09)	0.25
C4d	4 (4)	-0.08 (-0.27–0.12)	0.41
Interstitial fibrosis (ci)	87 (81)	-0.15 (-0.33–0.05)	0.13
Tubular atrophy (ct)	91 (84)	-0.16 (-0.35–0.03)	0.09
Vascular fibrous intimal thickening (cv)	36 (33)	-0.12 (-0.32–0.10)	0.28
GBM double contours (cg)	12 (11)	-0.18 (-0.36–0.03)	0.08
Mesangial matrix thickening (mm)	46 (43)	-0.07 (-0.27–0.13)	0.46
Hyaline arteriolar thickening (ah)	91 (84)	-0.06 (-0.26–0.14)	0.52
Inflammation in the area of IFTA (i-IFTA)	74 (69)	0.08 (-0.13–0.29)	0.45
PVI	13 (12)	0.35 (0.15–0.52)	0.0005 (***)

GBM, glomerular basement membrane; IFTA, interstitial fibrosis and tubular atrophy; PVI, polyomavirus-associated interstitial nephritis score; *** P<0.001; *P<0.05.

Supplementary Figures

Supplementary Figure S1 Dynamic changes in BK virus load following conversion from mycophenolic acid to mTOR inhibition in 13 kidney transplant recipients with BK virus-associated nephropathy.

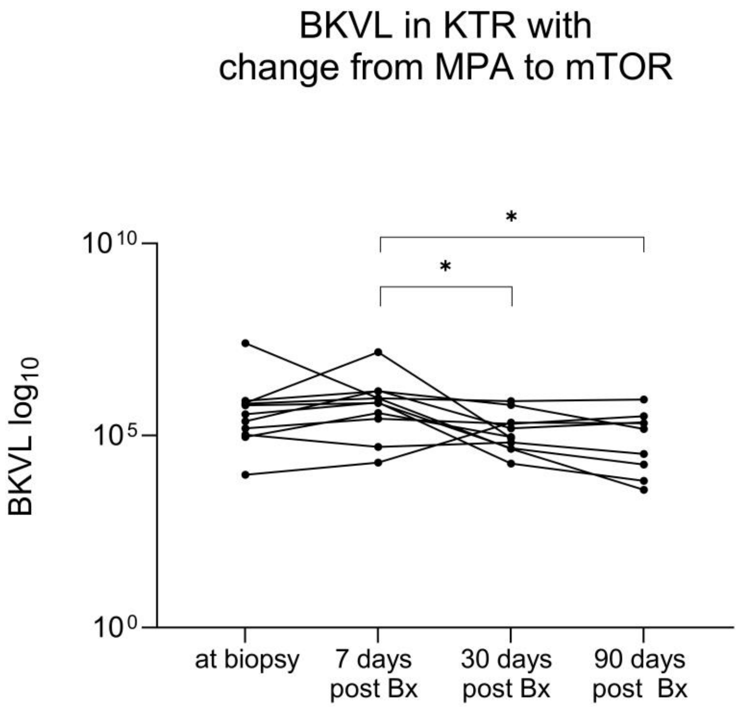

Kidney transplant recipients (KTR) with conversion from mycophenolic acid (MPA) to mTOR inhibitor following diagnosis for BKVAN (N=13) showed a significant decrease in BK viral load when comparing levels 30 and 90 days post biopsy to BK viremia 7 days post-biopsy (P=0.01 for both). The x-axis displays the different time points of BKV sampling while BKVL are presented on the y-axis. The lines connect different samples of the same patient.

BKVAN, BK virus-associated nephropathy; Bx, biopsy; KTR, kidney transplant recipients; MPA, mycophenolic acid; mTOR, mammalian target of rapamycin; BKV, BK virus; BKVL, BK virus load; * P<0.05
